# Distribution of Cathepsin K in Late Stage of Tooth Germ Development and Its Function in Degrading Enamel Matrix Proteins in Mouse

**DOI:** 10.1371/journal.pone.0169857

**Published:** 2017-01-17

**Authors:** Tao Jiang, Fen Liu, Wei-Guang Wang, Xin Jiang, Xuan Wen, Kai-Jin Hu, Yang Xue

**Affiliations:** 1 State Key Laboratory of Military Stomatology & National Clinical Research Center for Oral Diseases &Shaanxi Clinical Research Center for Oral Diseases, Department of Oral and Maxillofacial Surgery, School of Stomatology, the Fourth Military Medical University, Xi’an, P. R. China; 2 Department of Periodontology, School of Stomatology, the Fourth Military Medical University, Xi’an, P. R. China; 3 Department of Stomatology, Northwest Women's and Children's Hospital, Xi’an, P. R. China; 4 Department of Cardiovascular Surgery, Xijing Hospital, the Fourth Military Medical University, Xi’an, P. R. China; 5 Medical Unit, Troops PLA, Liaocheng, P. R. China; 6 Department of Oral and Maxillofacial Surgery, Dongfeng Hospital, Hubei University of Medicine, Shiyan, P. R. China; 7 Department of Prosthodontics, School of Stomatology, the Fourth Military Medical University, Xi’an, P. R. China; NIDCR/NIH, UNITED STATES

## Abstract

Cathepsin K (CTSK) is a member of cysteine proteinase family, and is predominantly expressed in osteoclastsfor degradationof bone matrix proteins. Given the similarity in physical properties of bone and dental mineralized tissues, including enamel, dentin and cementum, CTSK is likely to take part in mineralization process during odontogenesis. On the other hand, patients with pycnodysostosis caused by mutations of the *CTSK* gene displayedmultipledental abnormalities, such as hypoplasia of the enamel, obliterated pulp chambers, hypercementosis and periodontal disease. Thereforeitis necessary to study the metabolic role of CTSK in tooth matrix proteins. In this study, BALB/c mice at embryonic day 18 (E18), post-natal day 1 (P1), P5, P10 and P20 were used (5 mice at each time point)for systematic analyses of CTSK expression in the late stage of tooth germ development. We found that CTSK was abundantly expressed in the ameloblasts during secretory and maturation stages (P5 and P10) by immunohistochemistry stainings.During dentinogenesis, the staining was also intense in the mineralization stage (P5 and P10),but not detectable in the early stage of dentin formation (P1) and after tooth eruption (P20).Furthermore, through zymography and digestion test *in vitro*, CTSK was proved to be capable of hydrolyzing Emdogain and also cleaving Amelogenininto multiple products. Our resultsshed lights on revealing new functions of CTSK and pathogenesis of pycnodysostosis in oral tissues.

## Introduction

Cathepsin K (CTSK), also called lysosomal cysteine cathepsin K, contains a cysteine in its active site and functions mainly in lysosomes[1]. In the past, CTSK is believed to express predominantlyin osteoclasts, degrading bone matrix proteins, suchas type I collagen, osteonectin, and osteopontin inacidic conditions[2]. However, in recent years, CTSK has been reported to express in other cells, such as macrophages[3], bone marrow derived dendritic cells[4], fibroblasts[5]and intestinal goblet cells[6], playing an important role in extracellular matrix remodeling in different organs[7, 8], development and progression of cardiovascular diseases[9], as well as invasion of tumors[10]. The research and understanding of CTSK arealready far beyondthe limitation of osteoclasts and bone. However, it is still unclear whether CTSK is expressed in odontogenesis-related tissues.

Teethare also composed of three different kinds of hard tissues, including enamel, dentin and cementum. Despite different functions, these hard tissues share many similarities in structure and composition (Table 1). For instance, the mineralization processes of the dental hard tissues are similar or even identical to that of bone tissue. In clinical genetic studies, mutations in the *CTSK* gene have been found to cause pycnodysostosis,an autosomal recessive bone disease (OMIM 265800)[11]. The typical features of pycnodysostosis included increased bone density,short stature, osteolysis of the distal phalanges, frequent pathologic fractures[2], as well as dental abnormalities, such as hypoplasia of enamel[12–14], hypercementosis[15, 16], obliterated pulp chambers[16–18]and periodontal disease[14, 18]. Therefore, it is necessary to investigatethe expression feature and the role of CTSK in tooth development. In this study, we performed a systematic analysis ofexpression pattern of CTSK in odontogenesis and its role in degradation of enamel matrix proteins(EMPs).

**Table 1:** Comparison of the matrix composition in fourkinds of mineralized tissues.

	Enamel	Dentin	Cementum	Bone
**Mineral phase**	95 %	60~70%	About 50%	65% by the dry weight
**Organic matrix (>90 %)**	less than 1% (Amelogenin)	20~30% (Collagen І)	22% (Collagen І)	20~30% (Collagen І)
**Water**	4%	10%	About 30%	
**Hardness**	Enamel> Dentin≥ Bone>Cementum			

## Materials and Methods

### Ethical approval

This study was authorized by Ethic Committee, School of Stomatology, the Fourth Military Medical University, Xi’an,China.

### Animals

A total of twenty-five BALB/c mice at the embryonic day 18 (E18), post-natal day 1 (P1), P5, P10 and P20 were used (5 mice at each time point). Mandibles were isolated from the narcotized mice that were treated with 1% Pentobarbital Sodium (intraperitoneal injection).

### Tissue preparationand immunohistochemistry

Tissue preparation (includingfixation and demineralization), paraffin section, deparaffinization and rehydration were performed according to previously described methods [19].

Antigen retrieval, blocking of the activity of endogenous tissue peroxidase, non-specific binding, primary antibody [rabbit anti-mouse CTSK antibody (1:150, ab19027, Abcam, Cambridge, USA)] incubation, and immunohistochemistry staining were performed using Read-to-Use SABC-POD (Rabbit IgG) Kit (Wuhan Boster Biological Technology, Ltd., Wuhan, China). Immunoperoxidase staining (DAB kit, Beijing ComWin Biotech Co., Ltd., Beijing, China) was performed according to the manufacture’s protocol.

### Emdogain zymography

The zymography was performed using gels with 0.1% (w/v) Emdogain (Biora, AB Malmo, Sweden). Emdogain was dissolved in 1 mL/L acetic acid (Ruimeng, Jinan, China) to a concentration of 10 mg/mL. In brief, 3 μL(0.15 μg/μL) of Active human Procathepsin K protein fragment (ab157020, Abcam, Cambridge, USA) was mixed with 7 μL sterilized deionized water and then mixed with 2.5 μL of 5× SDS-sample buffer. Then electrophoresis was conducted with a Bio-RadMini-Protein system (Bio-Rad, Hercules CA, USA) with a constant voltage of 120 V at 4°C. After electrophoresis, in order to remove SDS, the gels were immersed in 2.5% (v/v) Triton X-100 for 1 hrs, washed twice in the incubation buffer (50 mM sodium acetate, 2.5 mM dithiothreitol (DTT), 2.5 mM EDTA, pH = 4.5), and then immersedin the incubation buffer at 37°C for 2, 6, 12, 24, 48 hrs, respectively. The gels were subsequently washed with water and stained in 45% methanol /10% acetic acid/water containing 0.25% Coomassie Brilliant Blue R-250 (MP Biomedicals, Shanghai, China).

### Digestion of AMELX by CTSK

Two micrograms (0.25 μg/μL) of Human Amelogenin, X-Linked (AMELX) full length protein (ab139212, Abcam, Cambridge, USA) with 0.15 μg or 0.025 μg of Active human Procathepsin K protein fragment (0.15 μg/μL) (ab157020, Abcam, Cambridge, USA)was incubated in 20 μL reactionsin incubation buffer as mentioned above for 0, 2, 4, 8, 12, 24, 36 and 48 hrs at 37°C. The reaction was ended by boiling for 5 min mixed with 5 μL of 5× SDS-sample buffer with reducing agents and analyzed by SDS–PAGE. Following electrophoresis, silver staining was performed according to the literature Handbook of Protein Technology [20].

## Results

### CTSK protein distribution in the developing mouse molars

**Fig 1:**
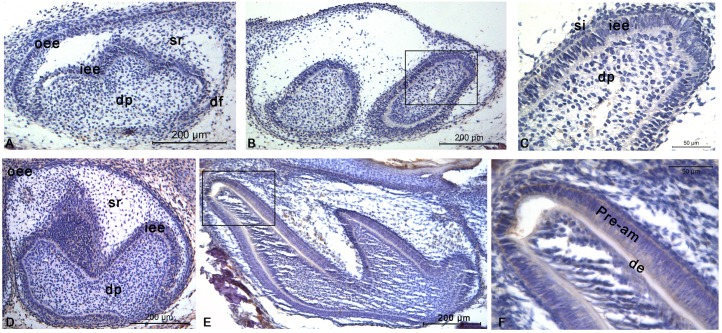
CTSK distribution in the developing molars of mouse at E18 and P1. Immunopositive staining for CTSK is not detectable in the second molar germ **(A)**, or the first molar germ **(B)** at E18. **(C)** Higher magnification of the frame in **(B)**. Immunopositive staining for CTSK couldn’t be detected in the second molar germ **(D)** at P1. However, weak staining could be observed in the first molar germ **(E)** at P1. **(F)** Higher magnification of the frame in **(E)**. Abbreviations: de, dentin; df, dental follicle; dp, dental papilla; iee, inner enamel epithelium; oee, outer enamel epithelium; Pre-am, pre-ameloblasts; sr, stellate reticulum.

At E18, the second molar was detectedduring early bell stage (Fig 1A), while the first molar was presentduring late bell stage (Fig 1B and 1C). However, no immunopositive staining for CTSK could be detected in either molar germ.

At P1, the second molar was seen during late bell stage (Fig 1D), while primary dentin formation and pre-ameloblasts differentiation could be seen in the first molar(Fig 1E and 1F). No immunopositive staining for CTSK could be detected in the second molar germ (Fig 1D), however, faint staining was observed in pre-ameloblasts in the first molar(Fig 1F).

**Fig 2:**
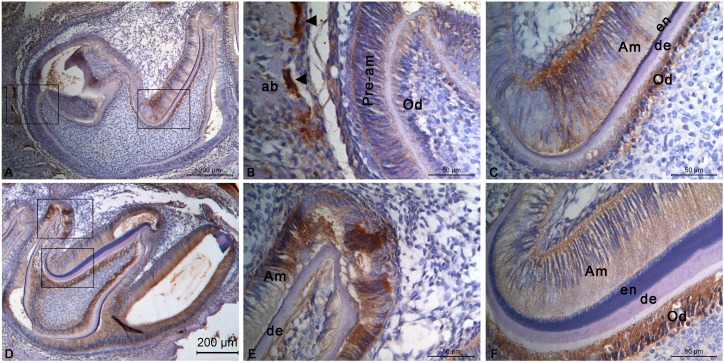
CTSK distribution in the developing molars of mouse at P5. Immunopositive staining for CTSK could be detected in both the second **(A)** and first **(D)** molars. **(B)** Higher magnification of the left frame in **(A)**. Osteoclasts were marked with ▲. **(C)** Higher magnification of the right frame in **(A)**. **(E)** Higher magnification of the upper frame in **(D)**. **(F)** Higher magnification of the lower frame in **(D)**. Abbreviations: ab, alveolar bone; Am, ameloblasts; de, dentin; en, enamel; Od, odontoblasts; Pre-am, pre-ameloblasts.

At P5, primary dentin and enamel could be seen in the second molar(Fig 2A–2C). CTSK labeling was absent from dental pulp tissues (Fig 2A). Weak staining of CTSK could be found in the pre-ameloblasts (Fig 2B). In contrast, the staining became very strong in the secretory ameloblasts and odontoblasts (Fig 2C). At the same time, mineralized alveolar bone began to form, with a strong staining of CTSK observed in osteoclasts on the bone surface (Fig 2B). In the first molar (Fig 2D–2F), primary dentin and secretory ameloblasts with intense reactivity could be seen at the tip of the cusps(Fig 2E), whilemineralized dentin and enamel as well as secretory ameloblasts and odontoblasts with strong staining of CTSK was found at more apical areas of the cusps (Fig 2F).

**Fig 3:**
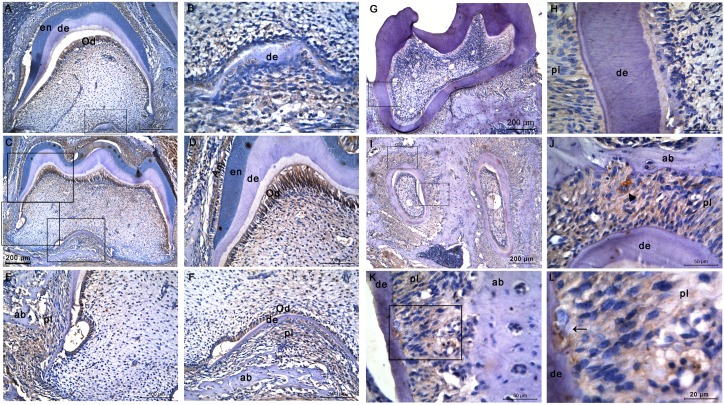
CTSK distribution in the developing molars of mouse at P10 and P20. During mineral matrix deposition, immunopositive stainings for CTSK could be detected in odontoblasts, mature ameloblasts, and in fibroblasts in periodontal tissues. **(A-F)** Molars of a mouse at P10.**(A)** The secondmolar. **(B)** Higher magnification of the frame in **(A)**showing newly formed furcation. **(C)** The first molars. **(D)** Higher magnification of the upper frame in **(C)**. **(E)** Higher magnification of the middle frame in **(C)**. **(F)** Higher magnification of the lower frame in **(C)**. **(G-L)** Molars of a mouse at P20. **(G)** Crown of the first molar. **(H)** Higher magnification of the frame in **(G)**. **(I)** Root of the second molar. **(J)** Higher magnification of the upper frame in **(I)**. Osteoclasts were marked with ▲. **(K)** Higher magnification of the lower frame in **(I)**. **(L)** Higher magnification of the frame in **(K)**. Odontoclasts were marked with ←. Abbreviations: ab, alveolar bone; Am, ameloblasts; de, dentin; en, enamel; Od, odontoblasts; pl, periodontal ligament.

At P10, furcation began to form in the second molar (Fig 3A and 3B)and also formed in the first molar (Fig 3C). Mineralized dentin and enamel could be seen in the crownsof both molars. Intense reactivity of the staining was detected in odontoblasts, either in the first molar (Fig 3D) or the second molar (Fig 3A), compared to a weaker staining in the mature ameloblasts. Simultaneously, mineralized alveolar bone could be seen in the distal side and under the furcation of the first molar, and a weaker immunostaining was seen in fibroblasts of periodontal tissues (Fig 3E and 3F).

At P20, the first (Fig 3G) as well as the second molar(Fig 3I) were fully developed and erupted. Periodontal ligament were connected to the root and the alveolar bone(Fig 3I). However, no clear cementum could be found on the root surface, outside the dentin(Fig 3J and 3K). A faint reactivity was observed in the fibroblasts in periodontal tissues(Fig 3H and 3J–3L), while a strong immunostaining was found in osteoclast(Fig 3J) as well as odontoclast (Fig 3L).

### The role of CTSK in degrading EMPs

**Fig 4:**
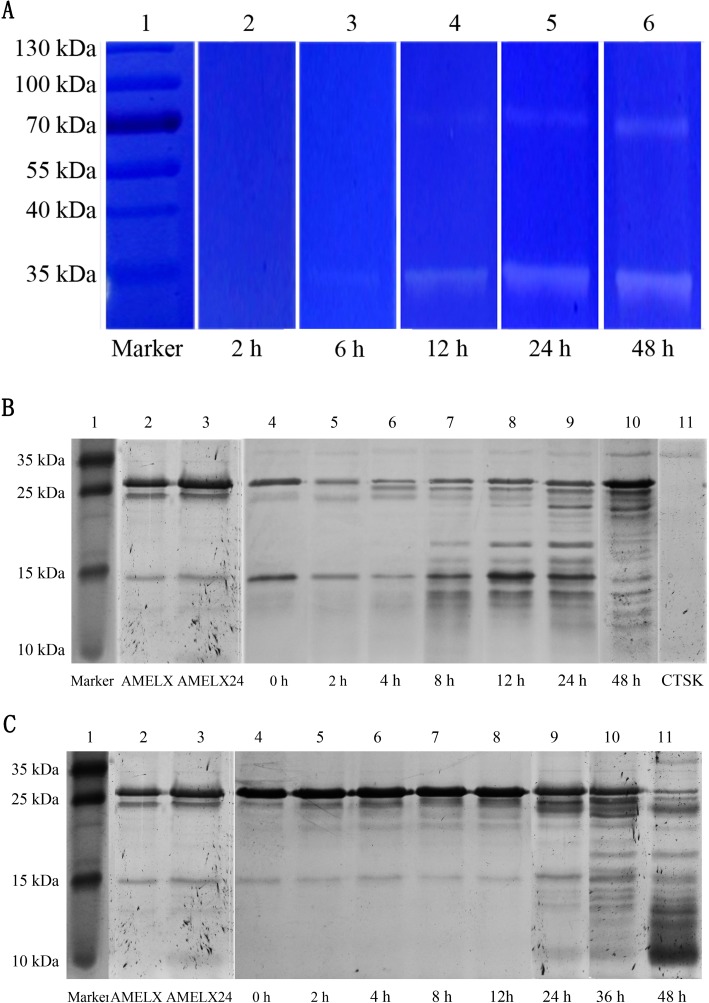
The role of CTSK in degrading enamel matrix protein. (A) Emdogain zymograms of CTSK. Areas of proteolysis appear as clear regions within the gel. Lane 1, marker; lane 2–6, the gels were immersed for 2, 6, 12, 24, 48 hrs respectively in the incubation buffer at 37°C. (B) Digestion of AMELX (2 μg) by CTSK (0.15 μg). Lane 1, marker; lane 2, standard aliquots of AMELX; lane 3, standard aliquots of AMELX incubated without CTSK at 37°C for 24 hrs; lane 4–10, digestion of AMELX by CTSK for 0, 2, 4, 8, 12, 24 and 48 hrs respectivelyat 37°C in the incubation buffer; lane 11, standard aliquots of CTSK. (C) Digestion of AMELX (2 μg) by CTSK (0.025 μg). Lane 1, marker; lane 2, standard aliquots of AMELX; lane 3, standard aliquots of AMELX incubated without CTSK at 37°C for 24 hrs; lane 4–11, digestion of AMELX by CTSK for 0, 2, 4, 8, 12, 24, 36 and 48 hrs at 37°C in incubation buffer, respectively.

CTSK was proved to be capable of hydrolyzing 0.1% Emdogain.The degradation began at the end of 6 hrs rather than 2 hrs. As time went on, the proteolysis regions became clearer and clearer. In addition to the bands near 35 kDa, clear bands also could be seen near 70 kDa at the end of 12, 24 and 48 hrs (Fig 4A). These bands may be resulted from a dimer of CTSK. On the other hand, CTSK was able to process AMELX into multiple cleavage products in both molarswith a ratio of 1:13 (0.15 μg: 2μg) (Fig 4B) and 1:80 (0.025 μg: 2μg)(Fig 4C). However, obvious cleavage products could be detected after 8 hrs with a higher ratio of CTSK, while after 24 hrs with a lower ratio of enzyme.

## Discussion

For the first time, we characterizedthe spatio-temporal expression patternof the CTSK protein during mouse molar development. The results showed that CTSK was mainly distributed in ameloblasts and odontoblasts during amelogenesis and dentinogenesis, while a weaker expression was found in periodontal fibroblasts. Additionally, we also firstly confirmed that CTSK could degrade EMPs.

Dental enamel is the hardest substance in the mammalian body[21]. However, during amelogenesis, soft enamel layer(82%protein in weight) is first formed[21–23]. Then, proteolytic degradation of EMPs (amelogenin, enamelin, and ameloblastin) is necessary for the full mineralization of enamel crystals[24]. At the end of the maturation stage, the fully formed enamel contains >95% mineral composition and <1% organic material[25]. To date, only two proteinases [kallikrein-related peptidase 4(KLK4) and matrix metalloproteinase 20(MMP20)] are believed to be secreted into the enamel matrix for degradingof the accumulated protein matrix[26]. However, mutations inKlk4or *Mmp20* or the EMPscannot be interpretedto be responsible for the whole spectrum of enamel phenotypes associated with amelogenesis imperfecta [27, 28], indicating that the existence of additional enamel-associated proteases. This study showed that CTSK was first present in trace amounts in pre-ameloblasts during the pre-secretory stage(Fig 1F). After that, a peak expression was then detected in secretory ameloblasts (Fig 2F),and continued to be expressed until the late maturation stage(Fig 3D). Though enamel phenotypeswere not yet describedin *Ctsk* knockout mice [29–31], enamel hypoplasiawas frequently reported in patients with pycnodysostosis due tovarious *CTSK* mutations [12–14]. Thus, the role of CTSK in the amelogenesis deserves further studies.

Through zymography analysis, we studied the degradation ability of CTSK on Emdogain, a commercially available product of EMPs. As a result, CTSK was proved to be capable of hydrolyzingEmdogain.The majority of Emdogain is amelogenin, which accounts for more than 90%.While amelogenin and its proteolytic cleavage products are also the most abundant EMPs in developing enamel[32]. Accordingly, CTSK and AMELX were incubated at different molar ratios. Unexpectedly, our results showed that CTSK was able to process AMELX into multiple cleavage products. This is the first evidence that CTSK was able to degrade EMPs.

On the other hand, ameloblasts were changed to ruffle-ended ameloblasts for proteinasessecretion during the maturation stage.Apparently, there has been a functional analogy between osteoclastsand ruffle-ended ameloblasts for many years[23](Table 2). One of the most important differences between the two types of cells was believed to be that ruffle-ended ameloblasts secreted MMPs and serine proteinases rather than cysteine proteinases[23]. However, the present study confirmed that CTSK is expressed in ameloblasts. This result was different from previous studies and added a new similarity between ruffle-ended ameloblasts and osteoclasts.

**Table 2:** Comparison between ruffle-ended ameloblasts and osteoclasts(summarized according to the description in Smith, 1998).

	Ruffle-ended ameloblasts	Osteoclasts
**Similarities**		
** Morphology**	ruffled borders	
** Enzymes on ruffled membrane**	H^+^-ATPase and carbonic anhydrase II	
** Resorb function**	resorb matrix proteins	
**Differences**		
** Origin**	long-lived epithelial cells	short-lived blood-derived cells (monocytes)
** Contact with mineralized tissue**	separated from the enamel by a basallamina	pressed directly against the mineralized tissue
**H**^**+**^**secretion and pHregulation**	despite reports of protonpumps, they probably release not H^+^ but the counterion, bicarbonate (the pH of maturing enamel does notdrop much below 6.0)	secrete H^+^ via V-ATPasesto lower localpH to as low as 4.0
Proteinases secretion*	secrete MMPs and serine proteinases, rather than cysteine proteinases (likecathepsin B) or aspartic (carboxyl) proteinases (likecathepsin D)	secrete lysosomal-type acid hydrolases, includingcathepsin B
** Calciumregulation**	assist movement of largeamounts of calcium from the blood into the maturingenamel layer	function indirectly to release calciuminto the blood

*: The present study confirmed that CTSK, a typical member of cysteine proteinase family and playing quite important role in osteoclast mediated bone resorption, was expressed in ameloblasts.

In addition to enamel, teeth are also composed of other two kinds of mineralized tissues, dentine and cementum. The process of mineralization is specific for each dental mineralized tissue. As mentioned above, enamel is unique in the body, involving specific proteins (amelogenin rather than collagen І), but not in elsewhere. Dentin, the major component of teeth, is a bone-like matrixproduced by odontoblasts [21, 33]. During the late bell stage in odontogenesis, odontoblasts are differentiated from ectomesenchymal cells in the dental papilla, and then secret extracellular matrix. CollagenI occupies 90% of the organic matrix along with non-collagenous proteins,including dentin sialophosphoprotein (DSPP), dentin matrix protein-1 (DMP1) and osteocalcin (OC)[33, 34]. After the so-called pre-dentin was formed, subsequent degradation or modification of the matrix proteins and final mineralization are followed to form mature dentin. Likewise, unmineralized osteoid is synthesized initially, and mineralized later[35]. So dentine belonged to a rather large group of connective tissue mineralization involving collagen and non-collagenousmatrix proteins[36]. Given the similarity in physical properties of bone and dentin (Table 1), CTSK could have an effect on dentin formation and regeneration. For the first time, we showedthat primary odontoblasts hadvery low level of CTSK protein during the early stage of dentin formation(Fig 2B). Then a strong immunostaining was detected in secretory odontoblasts during the dentin mineralization stage (Fig 2F),and persisted to the late stage of dentinogenesis (Fig 3D). However, CTSK was not found in the odontoblasts after tooth eruption (Fig 3H). On the other hand, patients with pycnodysostosis were also found to haveobliterated pulp chambers[16–18],suggesting that dysfunction of CTSK may result in impaired dentin matrix degradation.Whether CTSK plays a role in mineralization process of dentin needs a further study.

Cementum,covering the surface of tooth root,is a specific mineralized connective tissue. Its mineralization process is similar to that of bone[37]. It is well known that CTSK is a critical proteinase in osteoclast-mediated bone resorption[1]. Additionally, one of the most impressive dental features in pycnodysostosis patients is the thickened cementum[15, 16]. Unfortunately, the structure of cementum could not be observed in the current study (Fig 3I–3K). As previously reported, a genetically manipulated mouseis an excellent model tostudy odontogenesisbecause of its fast growth rate. However, with regard to cementogenesis,the rodent molar does not provide a good parallel for the human situation[38].

The present study also found a weak expression of CTSK in periodontal fibroblasts. This result was consistent with the previous study showing significantly immunoreactivity of CTSK in periodontal ligament of 12-week-old male mice[39]. However, its detail function remains unclear.

In conclusion,our results revealed that, during late tooth germ development, CTSK was mainly expressed by ameloblasts in secretory and maturation stages and odontoblasts in the mineralization stage of dentin. We also proved the capability of CTSKin hydrolyzingofEMPs. These findings shed lights on revealing new functions of CTSK and explaining the pathogenesis of pycnodysostosis. However, the exact role of CTSK in odontogenesis remains largely unknown.More studiesare needed to determinewhether this proteinase is secreted into the dental matrix and plays a role in dental mineralized tissue formation.
